# Chemical Exposures: PFOA Alters Liver Gene Expression

**Published:** 2006-08

**Authors:** Scott Fields

In the latest of a series of strikes against perfluorooctanoic acid (PFOA), the chemical has been found to affect gene expression in the livers of lab rats. PFOA is used in the manufacturing of fluorotelomers, which include nonstick substances such as DuPont’s Teflon^®^. PFOA is released when these fluorotelomers break down in the environment or the body. PFOA is stable in the environment, has been found in wildlife thousands of miles from an identifiable source, and bioaccumulates.

PFOA has been implicated in increasing in “bad” LDL cholesterol, while leaving “good” HDL cholesterol unaffected. Other studies have linked PFOA exposure to increased risk of stroke. PFOA is being phased out of use in the United States under a January 2006 agreement. DuPont will eliminate its PFOA use by 2015, and 3M has already phased it out of its Scotchgard™ line entirely. However, use of PFOA is increasing in Asia with the growth in industry there, especially in the Pearl River Delta of Southern China.

In the study, published in the January 2006 issue of *Toxicological Sciences*, Keerthi S. Guruge and colleagues exposed five groups of seven-week-old rats to daily doses of PFOA ranging from 1 to 15 mg/kg body weight. A control group received no PFOA. When the rats’ livers were tested the scientists found that the expression of more than 500 genes changed significantly at at least one dose level, and 144 were affected at all dose levels. The total number of genes affected peaked at the 10-mg/kg dose.

The largest category of genes affected were those that control how the liver transports and metabolizes lipids, especially fatty acids, says coauthor Paul K.S. Lam, a professor of biology at the City University of Hong Kong. Lam and Guruge—a senior scientist at Japan’s National Institute of Animal Health in Tsukuba—emphasize that these studies were conducted with hyperdoses of 100 to 1,000 times what might be found in environmental exposure.

Nonetheless, this work could be an important step toward explaining the increases in LDL seen with PFOA exposure, says Tim Kropp, a senior scientist for the nonprofit Environmental Working Group. “It starts to give you a clearer picture of what may be going on,” Kropp says. He adds that more animal studies are needed to put this work in context.

A related chemical, perfluorooctane sulfonate (PFOS), has been studied more extensively than PFOA, Lam says, but it’s important to look at the possible culprit itself. “There is a temptation for people to use existing data on PFOS for PFOA because there are some similarities in terms of the structure,” he says. “[But] no matter how similar they are, they are different.”

The team is now starting to look at how PFOA affects the kidneys, and they have expanded to the avian world with a chicken study to look for similar genetic effects. “If [the models] behave similarly,” Guruge says, “that means they must have some kind of common biomarkers.”

